# The relationship between lymphatic vascular density and vascular endothelial growth factor A (VEGF-A) expression with clinical-pathological features and survival in pancreatic adenocarcinomas

**DOI:** 10.1186/1746-1596-8-170

**Published:** 2013-10-18

**Authors:** Verônica A Zorgetto, Giórgia G Silveira, João Paulo Oliveira-Costa, Danilo F Soave, Fernando A Soares, Alfredo Ribeiro-Silva

**Affiliations:** 1 Departament of Pathology, Ribeirão Preto Medical School, University of São Paulo, 3900 Bandeirantes Avenue, Ribeirão Preto, São Paulo, Brazil; 2 Departament of Pathologic Anatomy A. C. Camargo Cancer Center São Paulo, São Paulo, Brazil

**Keywords:** Pancreatic cancer, VEGF-A, Angiogenesis, Lymphangiogenesis, Immunohistochemistry, Tissue microarray, Prognosis

## Abstract

**Background:**

Pancreatic cancer is a rare tumor with an extremely low survival rate. Its known risk factors include the chronic use of tobacco and excessive alcohol consumption and the presence of chronic inflammatory diseases, such as pancreatitis and type 2 diabetes. Angiogenesis and lymphangiogenesis, which have been the focus of recent research, are considered prognostic factors for cancer development. Knowing the angiogenic and lymphangiogenic profiles of a tumor may provide new insights for designing treatments according to the different properties of the tumor. The aim of this study was to evaluate the density of blood and lymphatic vessels, and the expression of VEGF-A, in pancreatic adenocarcinomas, as well as the relationship between blood and lymphatic vascular density and the prognostically important clinical-pathological features of pancreatic tumors.

**Methods:**

Paraffin blocks containing tumor samples from 100 patients who were diagnosed with pancreatic cancer between 1990 and 2010 were used to construct a tissue microarray. VEGF expression was assessed in these samples by immunohistochemistry. To assess the lymphatic and vascular properties of the tumors, 63 cases that contained sufficient material were sectioned routinely. The sections were then stained with the D2-40 antibody to identify the lymphatic vessels and with a CD34 antibody to identify the blood vessels. The vessels were counted individually with the Leica Application Suite v4 program. All statistical analyses were performed using SPSS 18.0 (Chicago, IL, USA) software, and p values ≤ 0.05 were considered significant.

**Results:**

In the Cox regression analysis, advanced age (p=0.03) and a history of type 2 diabetes (p=0.014) or chronic pancreatitis (p=0.02) were shown to be prognostic factors for pancreatic cancer. Blood vessel density (BVD) had no relationship with clinical-pathological features or death. Lymphatic vessel density (LVD) was inversely correlated with death (p=0.002), and by Kaplan-Meyer survival analysis, we found a significant association between low LVD (p=0.021), VEGF expression (p=0.023) and low patient survival.

**Conclusions:**

Pancreatic carcinogenesis is related to a history of chronic inflammatory processes, such as type 2 diabetes and chronic pancreatitis. In pancreatic cancer development, lymphangiogenesis can be considered an early event that enables the dissemination of metastases. VEGF expression and low LVD can be considered as poor prognostic factors as tumors with this profile are fast growing and highly aggressive.

**Virtual slides:**

The virtual slide(s) for this article can be found here: http://www.diagnosticpathology.diagnomx.eu/vs/5113892881028514

## Background

Pancreatic cancer is a rare tumor, according to the World Health Organization (WHO). The survival rate of patients is extremely low, and as such, it is considered as one of the most aggressive tumor types [1].

There are two types of cells in the pancreas, the exocrine cells and endocrine cells. The most common pancreatic tumors are originated from exocrine (epithelial) cells and trerefore are classified as adenocarcinomas. The adenocarcinomas account for up to 90% of pancreatic cancer cases. Pancreatic adenocarcinoma is considered rare before the age of 30 years and becomes more common after the age of 60 years.

The use of tobacco and its derivatives is considered the main risk factor for pancreatic cancer development as it increases the chance of pancreatic cancer development by three times compared with non-smokers. Other risk factors include the excessive consumption of alcoholic beverages, meat and fat and the presence of some diseases, such as chronic pancreatitis or type 2 diabetes (DM 2), as well as a family history of cancer [1].

Angiogenesis is considered as one of the most important factors for tumor development and progression. However, this process is also physiologically important [2]. Lymphangiogenesis, characterized by the growth of new lymphatic vessels, is absent in adults and is only present during the embryonic stage [3]. Angiogenesis and lymphangiogenesis can be found in pathological situations, such as chronic inflammation, wound healing and several neoplasms [3,4]. Both processes are induced by cytokines and growth factors, with the Vascular Endothelial Growth Factor family members (VEGFs) being the most important factors. While it is known that VEGF-C and VEGF-D are exclusively involved in lymphangiogenesis, studies have demonstrated that VEGF-A, which is present at a higher concentration and is associated with angiogenesis, also has an important role in tumor lymphangiogenesis [5,6]. The dynamic mechanisms of action of these growth factors and their receptors are still being investigated [3]. The activation of oncogenes due to genomic instability, as well as the action of some cytokines that increase the expression of VEGFs, also act as specific survival factors of endothelial cells, which support the development and progression of tumor cells [2,7].

During the neoplastic process, angiogenesis supplies nutrients to the primary tumor. At a later stage, the newly formed vessels provide routes for tumor cells to migrate, thus leading to metastasis in a process referred to as hematogenous spread. However, the dissemination of tumor cells via the lymphatic system is regarded as the main factor for the development of metastases in the regional lymph nodes and other distant organs [8,9].

In this way, both angiogenesis and lymphangiogenesis are considered important in cancer development. Knowing the angiogenic and lymphangiogenic profiles of a tumor may provide new insights that may enable targeted treatments according to tumor behavior [2,3,10,11].

The aim of this study was to evaluate the density of blood and lymphatic vessels, and the expression of VEGF-A, in pancreatic adenocarcinomas, as well as the relationship between blood and lymphatic vessel density (BVD and LVD) and the prognostically important clinical-pathological factors in pancreatic tumors.

## Methods

### Clinical characteristics of patients

In June 2012 we requested the medical records containing the clinical data of patients with pancreatic cancer diagnosed between 1990 and 2010 from the Medical Archive Service of our Institution. The samples had been collected from January 1990 to December 2010. The sampling procedure applied for diagnostic purpose was in accordance with current literature [12]. We excluded patients with a previous history of cancer or any type of oncologic treatment prior to the diagnosis of pancreatic cancer.

In the assessment of medical records, we obtained information concerning clinical data, such as age, gender and self-reported ethnicity. The following data were also retrieved: involvement of lymph nodes, presence of metastasis, disease-specific survival (DSS) and death. Factors that have been previously reported to contribute to the development of pancreatic cancer, such as DM 2, chronic pancreatitis and tobacco or alcohol consumption were also evaluated. Patients with insufficient clinical data from the medical records were excluded from the study.

The slides of these patients were retrieved from the Pathologic Service, and a pathologist (ARS) confirmed the diagnosis of pancreatic cancer, as well as the histological grade of the tumors.

One hundred patients who had sufficient clinical data and a sufficient amount of tumor sample in the paraffin blocks were included in the study.

The study was carried out in accordance with the 1975 Declaration of Helsinki and was approved by the local research ethics committee (Comitê de Ética em Pesquisa do Hospital das Clínicas da Faculdade de Medicina de Ribeirão Preto).

### Tissue Microarrays (TMA)

For the selection of tissues to be included in the TMA block, archived slides that were previously stained with hematoxylin and eosin were examined. All TMA processing was carried out in the Department of Pathologic Anatomy of A. C. Camargo Cancer Center (São Paulo, Brazil). Next, 1.1 mm^2^ cylindrical areas that corresponded to the most representative area of each lesion were removed from the paraffin blocks and transferred to a receiver block using a Manual Tissue Arrayer I (Beecher Instruments, Silver Spring, USA).

Each cylinder on the TMA slide represented one patient. From the TMA block, 3-μm histological sections were cut using a conventional rotary microtome (Microm HM315, Walldorf, Germany) and transferred to a TMA slide using the paraffin tape-transfer system (Instrumedics, Saint Louis, USA), according to manufacturer's instructions.

### Immunohistochemistry (IHC)

All samples retrieved had been fixed in 4% neutral formalin and embedded in paraffin. Of the 100 selected cases, only 63 contained sufficient material for traditional sectioning and counting of the blood and lymphatic vessels. The IHC reactions were developed using Reveal HRP (Spring – Code SPD-125). The paraffin sections were de-waxed in xylene and rehydrated through a series of graded alcohols. The sections were placed in 10 mM citrate buffer (pH 6) and were subjected to heat retrieval using a vapor lock for 30 minutes.

After antigen retrieval, the samples were allowed to cool for 15 minutes at room temperature. Endogenous peroxidase activity and proteins were blocked according to the manufacturer's protocol. The specimens were incubated at 4°C overnight with the following primary antibodies: D2-40 (Lymphatic Marker) (1:300, D2-40 clone, Biocare Medical, Concord, USA) for microvascular lymphatic counting, Endothelial Cell Marker (CD34) (1:200, QBEnd/10 clone, Novocastra Laboratories, Newcastle upon Tyne, UK) for blood vessels counting and VEGF (1:100, A-20 clone, Santa Cruz Biotechnology), which recognizes the isoforms with splicing at the 121, 165 and 189 amino acids.

After the incubation with primary antibody, the slides were incubated with the visualization system and then with the chromogen substrate, liquid diaminobenzidine (DAB). The slides were then stained with hematoxylin, dehydrated in a series of alcohols and permanently mounted with Permount polymer (Fischer, Fairlawn, NJ). Negative controls were prepared by the omission of the primary antibody.

We used a 10% cut off for the quantitative analysis of VEGF-positive neoplasic cells. As such, samples were considered positive when 10% or more cells showed cytoplasmatic VEGF expression, and samples were considered negatives if VEGF expression was absent or present in less than 10% of cells [13,14].

### Tumoral blood and lymphatic vessel counting

The blood and lymphatic vessel density was analyzed according to Lin et al. [8] and Weidner et al. [15]. The hotspots within the tumor sections were identified under low magnification (50 and 100x) using a light microscope. After the area of high microvascular density within the tumor was defined, the microvessels were counted individually under higher magnification (200 and 400x) with the Leica Application Suite v4 program (Leica Microsystems, Switzerland). Photomicrographs were also obtained using the same program. The lymphatic vessels were identified by immunohistochemical reaction with the D2-40 antibody, which resulted in the brown staining of the endothelial cells. The blood vessels were identified by reaction with the CD34 antibody, which resulted in the brown staining of the blood vessels. The BVD and LVD counts were performed separately on different slides.

### Statistical analysis

The statistical analysis of the relationship between the pathological and clinical data and the immunohistochemical results was performed by Fisher’s exact test and Chi-square test. Univariate analysis was conducted by the Kaplan-Meier method and Cox’s multiple regression was used for multivariate analysis. For both the clinical and pathological analyses and survival analysis, p<0. We 05 was considered statistically significant. All statistical analysis was performed using SPSS 18.0 (Chicago, IL, USA) software.

## Results

Of the paraffin blocks obtained from the Pathology Service, we excluded cases that did not have enough material for immunohistochemical reactions and/or for BVD and LVD counting or those for which the clinical information necessary for the research was not available. As such, this study was comprised of 100 cases for VEGF expression analysis by TMA and for epidemiologic study (clinical data) and 63 cases for the BVD and LVD analysis.

Of these 100 cases, 52% were men and 48% were women. With respect to self-reported ethnicity, 80% of the patients declared themselves white, 15.3% mixed race and 4.6% black. The ages at diagnosis ranged from 37 to 95 years (mean, 62 years). The median survival of this research population was 9.5 months, 67.6% of the patients had metastases, and 84.6% had died by the end date of the records search. Only 3.17% of patients had more than 4-year disease-specific survival.

### Independent prognostic factors

In the Cox regression analysis, the BVD and LVD did not have independent prognostic value for pancreatic cancer (p=0.885 and p=0.103, respectively). Among the data analyzed, only age (p=0.03), DM 2 (p=0.014) and chronic pancreatitis history (p=0.02) were statistically significant (Table 1).

**Table 1:** Independent prognostic factors

**Variable**	**Exp(B)**	**95% CI for Exp(B)**		**Sig.**
		**Lower**	**Upper**	
Age				0.074
Age (1)	0.483	0.082	2.837	0.42
Age (2)	0.131	0.021	0.825	0.03*
DM 2	0.228	0.07	0.743	0.014*
Chronic Pancreatitis	6.268	1.337	29.38	0.020*
VEGF-A	0.31	0.047	2.053	0.224
BVD	0.892	0.191	4.168	0.885
LVD	3.061	0.797	11.761	0.103

Multivariate analysis of clinical-pathological data, BVD, LVD and VEGF expression.

*A value of p<0.05 were considered significant.

### Blood and lymphatic vessel density analysis

**Figure 1:**
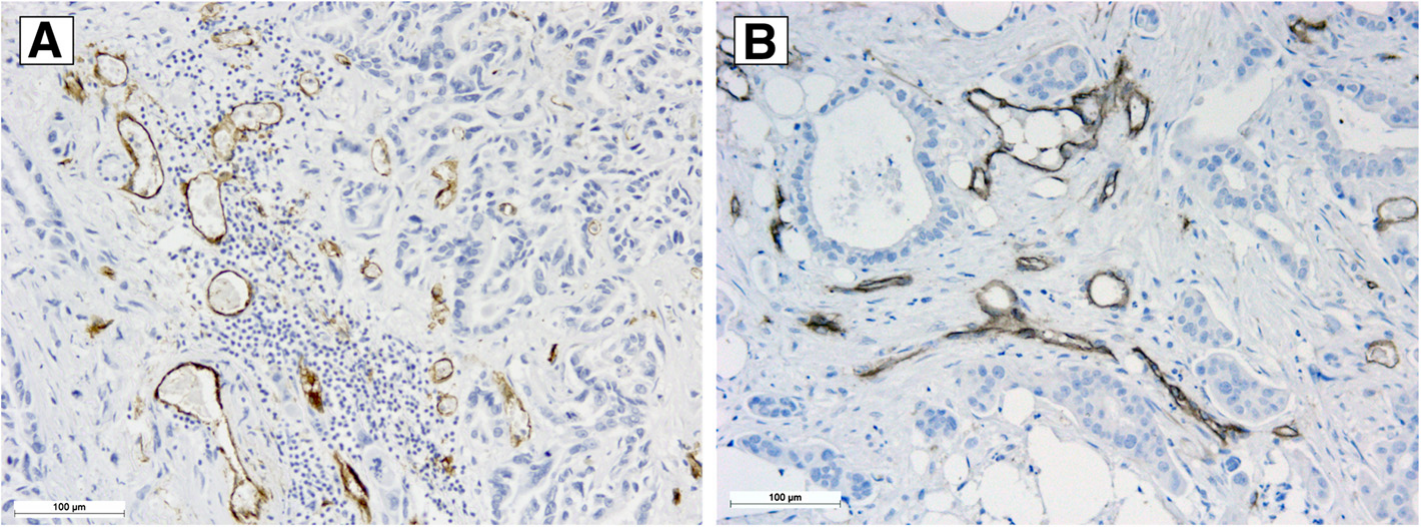
**Microphotograph of pancreatic adenocarcinoma. A**- Hotspot showing blood vessels stained by immunohistochemistry with CD34 antibody. **B**- Hotspot showing lymphatic vessels stained by immunohistochemistry with D2-40 antibody.

The average values of BVD and LVD were calculated from two hotspots from the same slide. The highest BVD and LVD values observed were more than 100 microvessels per field, while the lowest value found was 7 blood microvessels per field; there was an absence of neoformed lymphatic microvessels in this latter specimen. The data were dichotomized for the statistical analysis by identifying the average value for the blood and lymphatic vessels counted, which was 58 vessels per field for blood vessels and 15 vessels per field for lymphatic vessels. We considered values under 58 as low BVD and values above 58 blood vessels per field as high BVD and values lower than 15 as low LVD and values above 15 vessels per field as high LVD (Figure 1).

When the LVD and clinical-pathological parameters were compared, we found an inverse relationship between LVD and death events (p=0.002), while BVD had no significant relationship with death events (Table 2).

**Table 2:** Relationship between the clinical-pathological data and the lymphatic vascular density

**Characteristics**	**LVD**		**p-value**
	< 15 vessels	> 15 vessels	
**Gender**			0.793
Male	22	12	
Female	20	9	
**Age**			0.884
<60	20	11	
60-74	14	7	
>74	8	3	
**Alcohol consumption**			0.791
No	25	12	
Yes	16	9	
**Tobacco consumption**			1
No	26	13	
Yes	15	8	
**DM 2**			0.260
No	29	18	
Yes	13	3	
**Chronic Pancreatitis**			0.767
No	34	16	
Yes	7	5	
**Histologic Grade**			0.974
Well differentiated	12	6	
Moderately differentiated	22	11	
Poorly differentiated	5	3	
**Lymph node metastasis**			0.165
No	26	10	
Yes	12	11	
**Distant metastasis**			0.287
No	14	10	
Yes	28	11	
**Death**			0.002*
No	2	8	
Yes	40	13	

*A value of p<0.05 were considered significant.

### VEGF Expression

**Figure 2:**
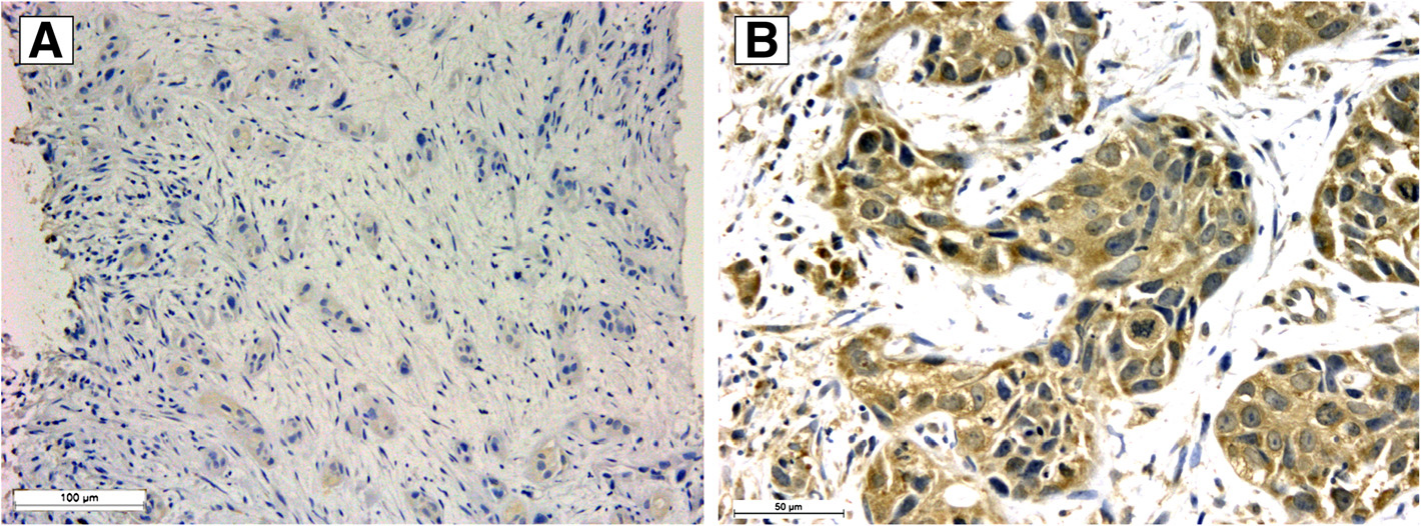
**Microphotograph of VEGF immunohistochemistry in pancreatic adenocarcinoma. A**- VEGF-negative specimen. **B**- VEGF-positive specimen.

Of the 100 cases analyzed by TMA, 71 specimens showed positive cytoplasmic VEGF staining, while 16 were negative, and 12 specimens were lost because of the technique (Figure 2).

### Correlation between BVD, LVD, and VEGF Expression and disease-specidic survival

**Figure 3:**
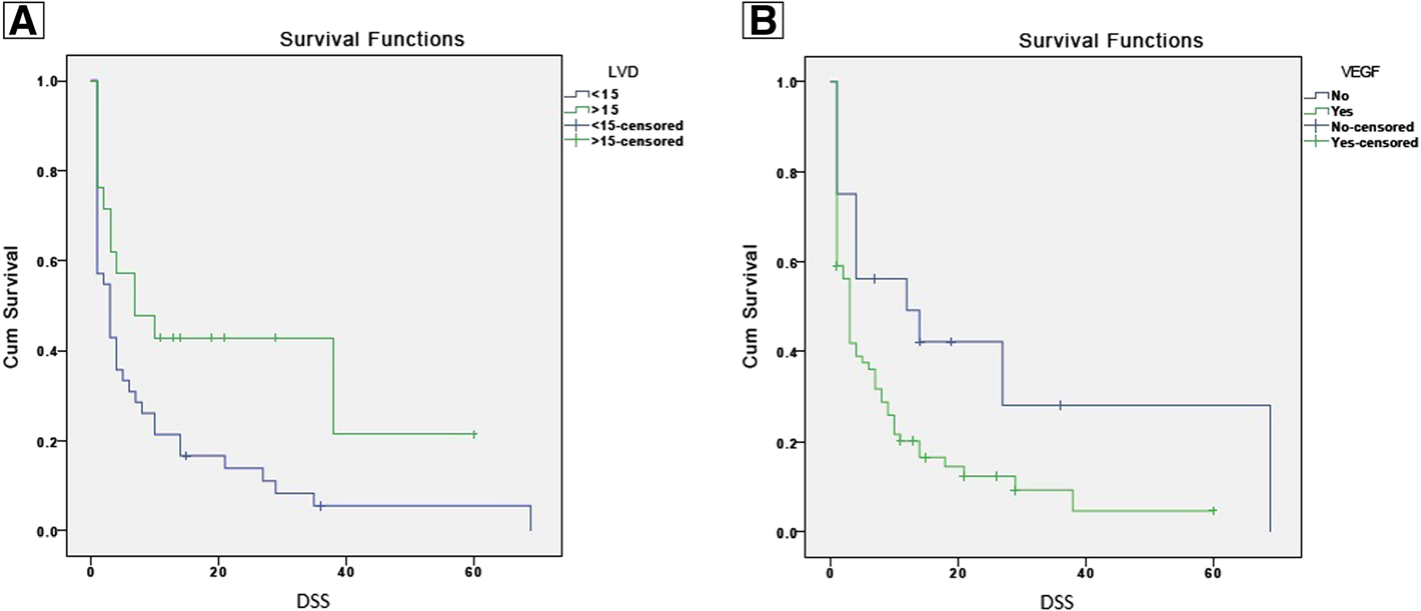
**Survival curves according to lymphatic Vessel Density (LVD) and VEGF-A expression. A**. Kaplan-Meyer plots analyzed by Mantel-Cox’s log-rank model. Time between diagnosis and death or the last data recorded is represented as a function of dichotomized lymphatic vascular density (p=0.021). DSS= disease-specific survival. **B**. Kaplan-Meyer plots analyzed by Mantel-Cox’s log-rank model. Time between diagnosis and death or the last data recorded is represented as a function of VEGF expression (p=0.023). DSS= disease-specific survival.

The disease-specific survival of the patients ranged from 1 to 69 months (mean, 9.5 months). Using the Kaplan-Meyer survival analysis with log-rank test, we found a significant association between low LVD and low patient survival (p=0.021) (Figure 3) and between VEGF expression and low patient survival (p=0.023) (Figure 3). However, we did not find any association between BVD and patient survival (p=0.175).

## Discussion

In our work we include only adenocarcinomas because they are far more comum than the pancreatic tumors of endocrine origins. However, it is worthwhile say that pancreatic tumors of endocrine origin are less aggressive than carcinomas, with distinct prognostic parameters [16]. The epidemiology for pancreatic cancer (adenocarcinoma) is heterogeneous, differing from one geographic region to another and in comparison to other types of tumors better studied, such as breast, prostate, lung and colorectal, little is known about the cellular and molecular prognostic factors of this disease. This knowledge gap is mainly due to the high aggressiveness of pancreatic tumors, which results in fewer than 5% of patients with 5-year survival after diagnosis. Additionally, pancreatic cancer presents with very subtle symptoms due to the retroperitoneal location of the organ [17]. Our study showed that in the studied population, only 3.17% of the patients achieved a survival rate of more than 4 years and that the average survival was 9.5 months, which is similar to the pancreatic cancer world statistics [17-20].

According to previous studies, men are more likely to develop pancreatic cancer, and the difference between the genders is higher in developed countries than in developing countries. The same trend was found in our study, in which 52% of the patients were men and 48% were women. Although pancreatic cancer is not among the ten most common cancers, its incidence tends to increase over time. In developing countries, this is mainly due to two primary reasons, increased life expectancy and the adoption of risk behavior, including high calorie and fatty food intake and consumption of tobacco and alcoholic beverages, at early ages [17,20].

Klöppel et al. [20] reported that 80% of pancreatic cancer cases are diagnosed in patients between 60 to 80 years of age. In our study, the average age of diagnosis was 65 years and ranged from 37 years to 95 years, which is consistent with previous reports in the literature.

With respect to self-declared ethnicity, 80% of the patients declared themselves white, 15.3% intermediate and 4.6% black. This is in contrast to other studies that have highlighted the black “race” as a risk factor for the pancreatic cancer development [17,20]. However, Takikita et al. [18] observed that in the cohort they studied, 75% of the tumors were well- or moderately differentiated and were significantly more common in whites than in other races. This is despite a lack of detailed information in the medical records of patients, as was the case in our study, and which is barrier for epidemiological population studies. Additionally, the high degree of mixing of the Brazilian population between three main parental populations, Native American, African and European, must be considered. As such, in scientific studies, the determination of ethnicity based on self-declaration or through the amount of pigment in the skin of the individual is debatable and is a very subjective classification, especially in populations with a high degree of miscegenation [21].

By multivariate analysis of the clinical-pathological data, we identified some independent prognostic factors for pancreatic cancer. An age higher than 74 years was an independent factor (p=0.03) because the higher the age of the individual, the greater the chance of developing neoplasms. The risk with respect to old age is already well known due to the duration allowing for the accumulation of mutations in the cells, mainly in the pool of stem cells, which multiply slowly. Over time, these cells acquire the necessary mutations to maintain a balance between them and the organ microenvironment [22]. Another significant prognostic factor was a history of DM 2 (p=0.014) and chronic pancreatitis (p=0.02), which demonstrated that they may be risk factors for pancreatic cancer. These data are in agreement with other several studies that have also identified DM 2 and chronic pancreatitis as risk factors [23-26].

Several studies have been conducted to clarify the relationship between DM 2, chronic pancreatitis and the risk of developing pancreatic cancer. Prizment et al. [25] showed that a particular single nucleotide polymorphism (SNP) associated with diabetes may also be associated with pancreatic cancer risk. Additionally, they observed that there were more cases of DM 2 in patients diagnosed with pancreatic cancer than among control patients who did not have a diagnosis of cancer. Another hypothesis, proposed by Braun, Bitton-Worms and LeRoith [23], was that insulin resistance, chronic inflammation and oxidative stress influence the development of pancreatic cancer because these processes are associated with both DM 2 and chronic pancreatitis. When the pancreatic beta cells are aberrantly hyperactive, the pancreatic tissue is exposed to high levels of insulin, which in turn has growth-promoting and mitogenic action, inducing cell proliferation. The tumor cells have a mechanism for capturing glucose that is independent from insulin, which confers a metabolic advantage. It is known that the high concentration of glucose in tissues leads to the formation of reactive oxygen species (ROS) that activate pro-inflammatory cytokines, which in turn, stimulate angiogenic factors such as VEGF [24]. By immunohistochemistry with CD34 and D2-40 antibodies, the blood and lymphatic vessels were identified, counted and tabulated. For statistical purposes, the values were dichotomized as low and high BVD or LVD (Figure 1). By comparing the LVD and the clinical-pathological parameters (Table 2), we found an inverse relationship between LVD and death events (p=0.002). No relationship was found between BVD and the clinical-pathological parameters. A similar result was found by Kaplan-Meyer survival curve analysis (Figure 3), in which low LVD was also related to poor patient survival and a bad prognosis (p=0.021).

The vascularization is considered an important step for tumor progression [27], so it is surprising the lack of association between BVD and prognostic parameters. However, the absence of a relationship between BVD and prognostic parameters found in our study was also previously reported by Kawauchi et al. [28] in synovial sarcomas. In squamous cell carcinoma of the larynx, Sullu et al. (2010) found that blood vessel density was significantly higher in high-grade tumors but did not correlate with other prognostic parmeters [29]. This lack of correlation between BVD and prognostic parameters could be due the tendency for pancreatic cancer to metastasize via the lymphangiogenic pathway. However, it also must be emphatizided that vascular diffusion density may also be involved in this process since only tumour cells within a distance less than 20 micro from the nearest neighbouring vessel are considered important for prognostic purpose [30].

The relationship between LVD and tumor behavior is controversial and has only been recently reported in the literature. Sleeman and Thiele [31] established a relationship between LVD and lymph node metastases in different types of tumors. Additionally, studies have demonstrated a positive relationship between peritumoral and/or intratumoral LVD, lymph node metastases and poor prognosis in squamous cell head and neck, endometrial and gastric tumors. On the other hand, no relationship was observed in pancreatic, ovarian and transitional cell bladder adenocarcinomas. Other reports have yielded conflicting results, such as in breast, lung, colorectal and oral esophageal cell tumors [32].

In pancreatic endocrine tumors, Rubbia-Brandt et al. [33] found a relationship between LVD and tumor clinical behavior. However, there was no relationship between LVD and the presence of lymph node metastases. The expression of VEGF-C, an inducer of lymphatic vessels, was lower in benign tumors than in well-differentiated, malignant tumors. These data show that high LVD is not required for the proliferation of lymphatic vessels and that the malignant transformation of the tumor leads to the induction of pro-lymphangiogenic factors. Additionally, despite the tendency for an increased number of lymphatic vessels in the tumor over time, the invasion of tumor cells into the lymph capillaries occurs early in the tumorigenic process [33]. Similarly, Sipos et al. [34] reported that the LVD in malignant tumors was increased compared to that found in normal pancreas or in chronic inflammation. However, the intratumoral LVD was low, and the lymph vessels showed decreased proliferation, rather than being stimulated due to the presence of inflammatory factors. As such, functional intratumoral lymphatic vessels are not required during lymphatic invasion, and their loss of function can be caused by several different mechanisms [35].

Because we observed an inverse relationship between LVD and survival, we hypothesized that low LVD is due to the accelerated growth of the tumor and the eventual loss of function of the lymphatic vessels that were present. Accordingly, tumors with low LVD are more likely to be fast growing and of a more advanced stage, which would explain the low survival rate of these patients. The function of these vessels could have been lost because of the internal pressure caused by rapid tumor growth, which would lead to the collapse of the vessels, the invasion of tumor cells in the lymph vessels or other mechanisms that have yet to be elucidated [35]. Some studies have suggested that despite the low intratumoral LVD, one can still find a high peritumoral LVD. These may be the vessels through which lymphatic metastasis occurs, resulting in the spread of tumor cells to the regional lymph nodes and, in the case of pancreatic tumors, the liver [33,36].

In agreement with the research by Yin et al. [37], which demonstrated the importance of the lymphangiogenesis in the evolution of diabetes and pancreatic inflammation, our work found these diseases to be independent prognostic factors for pancreatic cancer. Furthermore, the formation of new lymph vessels is an early process that develops before tumorigenesis, and as the tumor grows, the lymph vessels are inhibited or disabled. Our study also reports the same prognostic importance of LVD that was found by Wang et al. [38].

Of the 100 pancreatic tumor cases that were used to construct the TMA and subsequently analyzed by immunohistochemistry for VEGF expression, 71% showed cytoplasmic VEGF expression. By Kaplan-Meyer survival curve analysis, the expression of VEGF in tumors proved to be significantly associated (p=0.023) with poor prognosis and a reduction in patient survival time.

It is known that the VEGF family of proteins contributes to the development of new blood vessels through the process of angiogenesis, which was widely studied and discussed by Folkman and reviewed by other researchers. Angiogenesis leads to the spread of tumor cells by hematogenous routes and, consequently, to metastasis [39-42]. The involvement of other organs by metastases significantly decreases the patient's survival. Additionally, the overexpression of VEGFs by the tumor or cells in the extracellular matrix leads to the rapid growth of the tumor mass because these factors have mitogenic properties that stimulate DNA synthesis in endothelial cells and provide better nutrition and tumor oxygenation through the formation of new blood vessels [43].

The family of VEGFs is composed of VEGF-A, VEGF-B, VEGF-C, VEGF-D, VEGF-E, VEGF-F, and placental growth factor (PlGF). VEGF-A was the first to be discovered and, therefore, was originally named VEGF. This factor was directly linked to angiogenesis that occurs in physiological processes, such as embryonic development, and in pathological processes, which include inflammation, wound healing and tumor growth. The function of VEGF-B, which is expressed in skeletal and cardiac muscle and brown adipose tissue, is not understood. However, unlike VEGF-A, its expression is not stimulated by low temperatures or by hypoxia. VEGF-C and-D are linked to lymphangiogenesis and, unlike VEGF-A, are only expressed in lymphatic endothelial cells in physiological situations. The most recently discovered factors are VEGF-E, which is encoded by a virus, and VEGF-F, which is expressed in the venom of a snake [43].

In our research, we used a VEGF antibody that, according to the manufacturer, has affinity for the protein isoforms with splicing in amino acids 121, 165 and 189, which correspond to VEGF-A [44,45]. The relationship that was observed between the presence of VEGF-A and low survival can be accounted for by the mitogenic activities of VEGF-A, which contribute to the rapid growth of the tumor and, consequently, poor prognosis.

The independent prognostic factors identified in our study, advanced age and a history of DM 2 or chronic pancreatitis, are supported by the relationship between inflammation and the increased expression of VEGF. During inflammatory diseases, the chronic high concentrations of insulin, ROS, cytokines and inflammatory mediators, such as TNF-α and COX-2, lead to an increase the expression of pro-angiogenic factors, the most important being VEGF-A [23,24].

Even though VEGF-A is directly linked to angiogenesis, recent studies have shown that this factor is also involved in lymphangiogenesis during tumor development. The biological effects of the VEGF family members occur through the interaction of the factors, which are secreted by tumor cells or matrix inflammatory cells, with transmembrane receptors located in the vascular and lymphatic endothelial cells. The interaction of the ligand with receptor leads to dimerization and the autophosphorylation of the receptor intracellular domains that, in turn, generates a cascade of reactions involving proteins related to cellular survival and division. VEGF-A can interact with the receptors VEGFR-1, VEGFR-2 and the neuropilins 1 and 2. However, it is through interaction with the VEGFR-2, found specifically in lymphatic endothelial cells, that lymphangiogenesis is induced [43].

VEGF-A can promote lymphangiogenesis either indirectly or directly. Indirectly, it can recruit inflammatory cells that, in turn, will produce VEGF-C and VEGF-D, which are known to promote lymphangiogenesis. Directly, VEGF-A will interact with its receptor, VEGFR-2, which is present in pre-existing lymphatic vessels, activating them [5]. According to Wirzenius et al. [46], while the binding of VEGF-A to VEGFR-3 affects the spreading of the vessel network, its binding to VEGFR-2 produces an increase in lymphatic vessels size, making them able to drain the interstitial fluid of the tumors. In this way, VEGF-A promotes the development of an ideal pre-metastatic niche for the initiation of metastases [6].

VEGF expression is increased both at the transcription and translation levels in pancreatic tumor tissues, compared to surrounding non-tumoral tissue. Its expression, according to Liang et al. [47], is related to tumor size, stage and lymph node metastases, demonstrating it as an important prognostic marker of tumor behavior.

In our study, we demonstrated that the LVD is inversely related to survival, while the expression of VEGF-A is directly related to survival. In accordance with the current literature, we postulate that the expression of VEGF-A is an early event in the development of cancer. Inflammatory processes, which are known to increase the likelihood of cancer development, also promote the overexpression of VEGF-A [24,25,48]. The higher the expression of VEGF in the tumor during its development, the faster the growth of the tumor. In turn, this causes the previously developed lymph vessels to be inactivated by the increased internal pressure of the tumor and, consequently, they will collapse and be destroyed [35].

## Conclusions

We conclude that advanced age and a history of type 2 diabetes mellitus or chronic pancreatitis are independent prognostic factors for the development of pancreatic cancer. We also conclude that the expression of VEGF-A and low LVD may contribute significantly to poor prognosis because of increased tumor aggressiveness and low patient survival. We add new evidences in literature that lymphatic vessel density is related to survival in patients with pancreatic carcinoma and is relationship with VEGF-A expression may be important for future therapeutic strategies.

## Abbreviations

WHO: World Health Organization; INCA: Instituto Nacional do Câncer; DM2: Diabetes mellitus type 2; VEGFs: Vascular endothelial growth factors; DSS: Disease specific survival; TMA: Tissue microarray; IHC: Immunohistochemistry; BVD: Blood vessel density; LVD: Lymphatic vessel density; DNA: Deoxyribonucleic acid; PlGF: Placenta growth factor; TNF-α: Tumoral necrose factor-α; COX-2: Cyclooxygenase-2; VEGFR: Vascular endothelial growth factor receptor; ROS: Reactive oxygen species.

## Competing interests

The authors declare that they have no competing interests.

## Authors’ contributions

VAZ: design and intellectual content definition, data acquisition and analysis and preparation of the manuscript. GGS: design and intellectual content definition, data analysis and critical review of the manuscript. JPOC: statistical analysis. DFS: critical review of the manuscript. FAS: coordination of TMA slide preparation. ARS: anatomopathologic examination of the samples, research coordination and critical review of the final manuscript. All authors read and approved the final manuscript.
